# Supplementary material for: Chitosan–Azide Nanoparticle Coating as a Degradation Barrier in Multilayered Polyelectrolyte Drug Delivery Systems
Source: Biomolecules. 2019 Oct 5;9(10):573. doi: 10.3390/biom9100573 (PMC6843188; doi:10.3390/biom9100573)
Supplement: Supplementary file 1 [file biomolecules-09-00573-s001.pdf]

biomolecules

Supplement to

Chitosan-azide nanoparticle coating as degradation barrier in multilayered polyelectrolyte drug delivery systems

Steffen Sydow [1], Armin Aniol [1], Christoph Hadler [1] and Henning Menzel [1,*]

[1] Institute for Technical Chemistry, Braunschweig University of Technology, 38106 Braunschweig, Germany
* Correspondence: h.menzel@tu-bs.de; Tel.: +49-531-391-5361

Supplementary Materials:

Purification of Chitosan: Chitosan (DDA 75-85%) was purified by a method according to GAN ET AL. Chitosan was added to a 1 M NaOH and stirred at 70°C for 2h. Chitosan flakes were filtered off, washed with deionized water and were dissolved in 1% (w/v) acetic acid. Finally, the purified chitosan was obtained by dialyses of the filtrate (cutoff 10 kDA) against 0.1 M NaCL solution and deionized water following by subsequent lyophilization.

Azide-"blocking"-middle layer

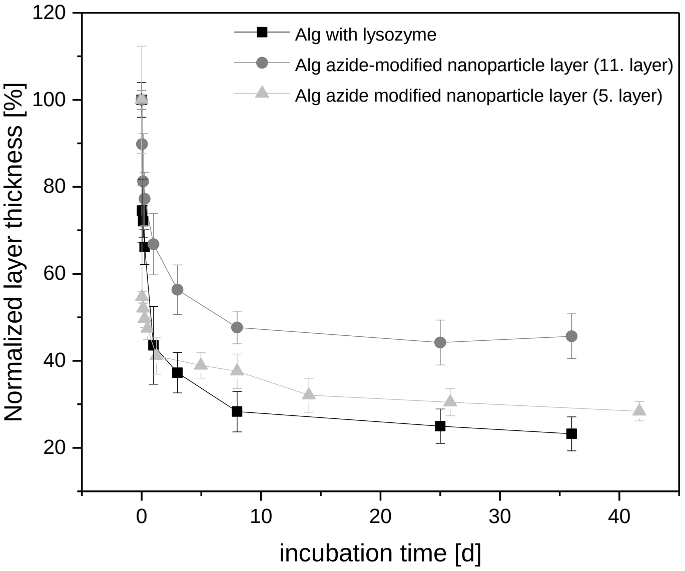

Figure S1: Degradation of CS(17)-TPP multilayer system (Alg) in PBS-puffer at 37°C. Lysozyme has been added to reach a concentration of 1,5 µg/mL. For CS-Az retarding, the 11th top layer was deposited by dipping in 1 mg/mL CS-Az-TPP NP solution and subsequently crosslinking under UV-light. For the intermediate blocking layer CS-Az-TPP NPs were deposited at 5th layer. Lysozyme was also added to the CS-Az experimental group. N > 3.

© 2019 by the authors. Submitted for possible open access publication under the terms and conditions of the Creative Commons Attribution (CC BY) license (http://creativecommons.org/licenses/by/4.0/).
